# Formation of Unique Placental Seed Capsules in the Maturation Process of the Tomato Fruit

**DOI:** 10.3390/ijms231911101

**Published:** 2022-09-21

**Authors:** Inna A. Chaban, Alexander A. Gulevich, Ekaterina N. Baranova

**Affiliations:** 1 Plant Cell Biology Laboratory, All-Russia Research Institute of Agricultural Biotechnology, Timiryzevskaya 42, 127550 Moscow, Russia; 2 Plant Cell Engineering Laboratory, All-Russia Research Institute of Agricultural Biotechnology, Timiryazevskaya 42, 127550 Moscow, Russia; 3 Plant Protection Laboratory, N.V. Tsitsin Main Botanical Garden of Russian Academy of Sciences, Botanicheskaya 4, 127276 Moscow, Russia

**Keywords:** tomato fruit, seed development, seed capsule, seed capsule shell, seed coat, elongated cells, calcium oxalate crystals, outer layer cracking, ribbon-like hairs

## Abstract

The morphological and anatomical study of the seed formation features in a juicy tomato fruit was carried out. The ovules, which form on the placenta, have been shown to be gradually enveloped by the protrusions of placental tissue that arises simultaneously with them. As a result of this process, each seed is enclosed in an individual capsule. These seed capsules have been shown in vivo to be airtight and air-filled. Tomato seeds, as has been shown in this study, develop inside these capsules until the full maturity of the fruit and do not come into contact with the detached and moldered cells of the placenta protrusions, which convert into a gel (pulp). Using scanning electron microscopy, it was possible to reveal the details of a ribbon-like “pubescence” formation of the tomato seed, as well as to understand the mechanism of cracking of the outer layer cells in the seed coat, associated with the detection of calcium oxalate crystals in these cells. The unique outer layer of the tomato seed coat seems to play the role of a scaffold that maintains a constant volume of the protective capsule.

## 1. Introduction

Angiosperms show a wide variety of seeds with different anatomical features. This diversity reflects their main features of development and evolution, such as the distribution system, metabolism, and adaptation to the environment [1].

Fruits are specialized structures designed to protect the developing angiosperm seeds and distribute them properly [2]. Fruits and seeds depend on each other to varying degrees in the process of development and performance of their biological function. By its structure, the fruit is a complex organ, consisting of several tissues with different purposes. The fruit is developed from the ovary. The size of the ovary and, accordingly, the fruit, depend on the number of accreted carpels that formed the ovary. The pericarp of the fruit is formed from the walls of the ovary. As a rule, it consists of three parts—exocarp, mesocarp and endocarp. Depending on the type of the pericarp structure, the fruits are divided into dry and juicy (fleshy) [2,3]. Plants from the *Solanaceae* family have different types of both dry and juicy fruits. Usually, the fruit of *Solanaceae* is a berry (*Solanum nigrum* L.; *Solanum tuberosum* L.; *Solanum lycopersicum* L.; *Atropa belladonna* L.) or capsule (*Nicotiana tabacum* L.; *Hyoscyamus niger* L.; *Datura stramonium* L.). At the same time, there are differences between different types of fruits and within them. This anatomical complexity of plant organs is closely related to changes in their metabolism during the development of individual tissues, since different plant organs, tissues or cells are characterized by a specific structure and distribution of metabolites.

Seed maturation of most *Solanaceae* occurs in the air ambience, and only in fleshy berries like tomato are the seeds inside the ripening fruit in a humid environment, since they are immersed in the tissue of an overgrown placenta, the cells of which undergo maceration and turn into a gel. Tomato fruits are most widely used as a model object for various basic studies. Most molecular and biochemical studies of tomato fruit development involve the entire fruit or just the pericarp. Studies of various specific tissues remain rare. However, each tissue has at least one specific role. In a number of studies, the tissue specificity of the tomato fruit pericarp was emphasized by molecular genetic studies based on manual tissue dissection [4,5,6]. However, only a few studies have characterized all tomato fruit tissues separated by manual dissection. The composition of each tissue has been shown to change during fruit development. In other words, each tissue follows its own lineage during fruit development [7]. Therefore, a proper understanding of the fruits and seeds anatomy, as well as their dynamic evolution, is crucial to support various kinds of research, including those related to fruit metabolism and genetics [8].

The seeds in the juicy fruits of most *Solanaceae* have a relatively smooth surface. However, only in tomato do they have a characteristic expansion of the integumentary epidermis cells, leading to the formation of a visible pubescence caused by a violation of the integrity of the cell walls in this layer. And when mature, these seeds are in the mucilaginous shell. In many works, the question becomes as follows: what in a ripe tomato fruit inhibits the germination of seeds located in a cavity filled with cell sap that appeared as a result of cell maceration and the destruction of cell walls to the mucilage state?

There is a generally accepted opinion that this liquid (pulp) contains substances that delay the germination of seeds, for example, organic acids, sugars, and unfavorable ratio of hormones. In addition, it has been suggested that the osmotic environment in tomato fruit tissues plays an important role in maintaining development and preventing premature seed germination [9]. In the same paper, attention was drawn to the shells surrounding the locular tissue, and it was suggested that they are also able to prevent the germination of developing tomato seeds, along with the pulp. For this reason, tomato seeds are usually washed and even subjected to technical polishing to get rid of the inhibitory effect of the pulp (gel) surrounding the seeds. However, until now this issue has remained open and unclear, which was the reason for us to undertake the present study of the shells enveloping the tomato seed.

The aim of the work was a detailed anatomical study of a number of not fully understood issues related to the morphology, formation and functioning of some structures and tissues of the tomato fruit and seed.

## 2. Results

**Figure 1:**
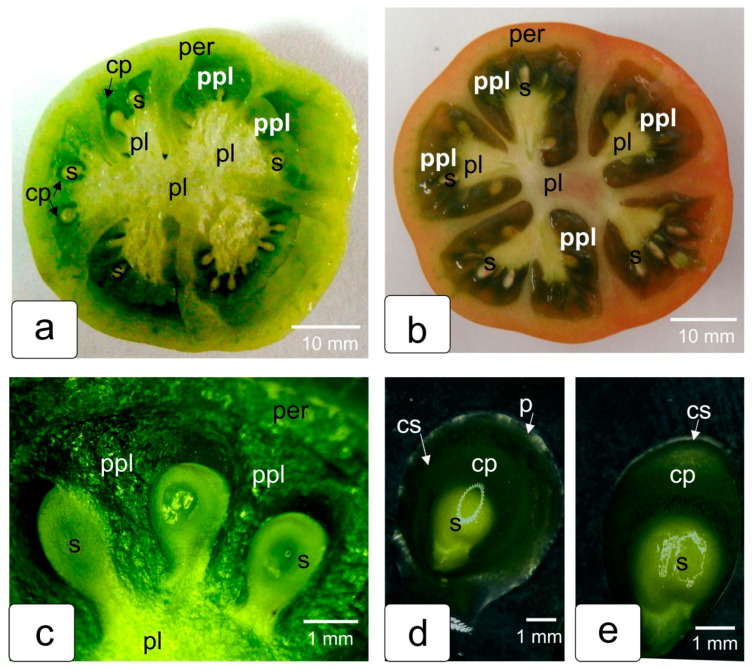
The internal structure of the tomato fruit at two stages of ripening—green fruit (**a**) and mature red fruit (**b**); transverse cut. The fruit is divided into several locular cavities forming the protrusions of placental tissue (ppl) with seeds (s) enclosed in them. Each seed is in an individual capsule and surrounded by parenchyma tissue of placenta (**a**,**c**). In the red fruit, the seeds are embedded in a jelly-like mass (gel) formed from the destroyed parenchyma cells of the lateral placenta (**b**). The seeds are easily removed from the gel together with the seed capsules and with the gel remnants on the seed capsule surface (**d**), next to it is the capsule with the seed, carefully cleaned of the gel (**e**). Designations: cp—capsule; cs—capsule shell; per—pericarp; pl—placenta; s—seed; ppl—protrusions of placental tissue.

A tomato fruit with multiple locules has axile placentation. Lateral branches extend from the central placenta into each locular cavity (seed chamber). Ovules are formed on these placentas (Figure 1a). After fertilization, in parallel with the development of ovules, tubercles of the protrusions of placental tissue are laid between them (Figure 1c). This placenta envelops each ovule and separates them from each other. As a result of this process, each ovule is enclosed in an individual capsule. This is clearly seen in the cross section of a green tomato fruit (Figure 1a,c). Each capsule is surrounded by multicellular parenchyma tissue in the protrusions of placental tissue. In a cross section of a red, ripe tomato fruit, the seeds appear to be completely embedded in a jelly-like mass resulting from maceration of the cells of the secondary lateral placenta (Figure 1b). But if the seed is carefully removed from this mass, it can be seen that the capsule is preserved and completely isolates the seed from the surrounding so-called gel (Figure 1d,e).

**Figure 2:**
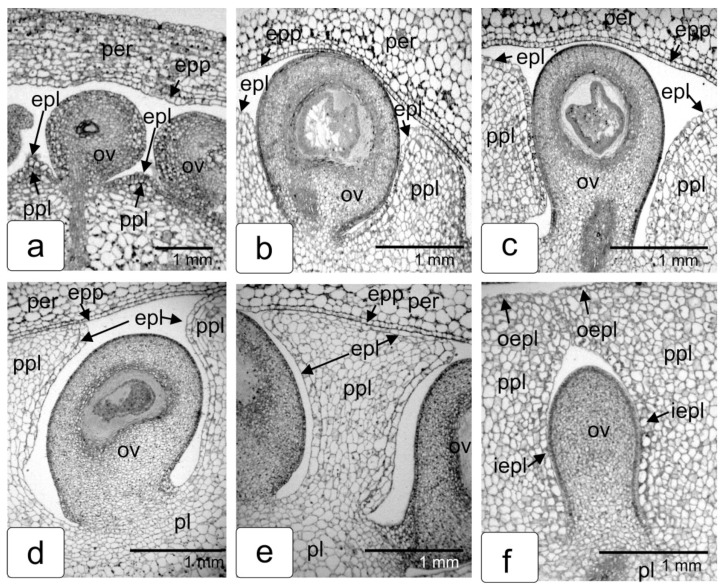
Structural illustration of the process of seed capsule formation during tomato fruit development. Appearance of outgrowths of the lateral placenta between the developing ovules (**a**); elongation of these outgrowths towards the pericarp (**b**,**c**); subsequent encirclement of each ovule by the growing placenta (**d**–**f**). The inner parts of the epidermis of the placenta surrounding each ovule form a capsule shell (**f**). Designations: epl—epidermis of placenta; epp—epidermis of pericarp; iepl—inner epidermis of protruding placenta tissue; int—integument; oepl—outer epidermis of protruding placenta tissue; ov—ovule; per—pericarp; ppl—protrusion of placenta tissue.

We carried out a detailed anatomical study of the various stages of tomato fruit formation in order to follow the entire process of formation of these placental capsules, individual teach ovule. The steps of this process are shown in Figure 2a–f. Figure 2a clearly shows the projections of the placenta between the developing ovules. As the ovules develop, placental protrusions between them grow towards the pericarp (Figure 2b,c.) In fact, these protrusions are part of a single placenta having parenchyma cells and epidermal tissue gradually filling the space between the developing seeds and forming a space isolated by the placental epidermis around the individual seed. This becomes obvious if we imagine the processes occurring inside the fruit in volume, and not on a flat section. The parenchyma tissue of the placenta consists of rather small cells and is surrounded by the specialized epidermal tissue separating the parenchyma cells from the air cavity of the internal space of the fruit and seed surface (Figure 2e). The epidermis is made up of larger, tightly connected cells. When growing, the placenta envelops each ovule and then closes over them. As a result of this, the protruding parts of the inner epidermis of the placenta are closed, forming a single shell of a closed chamber—a capsule for each ovule (f). The outer parts of the epidermis of the protrusions of placental tissue also join to form a single outer epidermis of the placental protrusion, which restricts the pulp (fruit pulp) at the border with the inner epidermis of the pericarp under ripening (Figure 2e,f). It is in this way that individual formations are formed in the tomato fruit—hermetic capsules for each seed. When the placental tissue in the ripe fruit breaks down and turns into a gel (jelly-like mass), the epidermal shell of the capsule is preserved and hermetically seals the seed until the tomato fruit is fully ripe.

**Figure 3:**
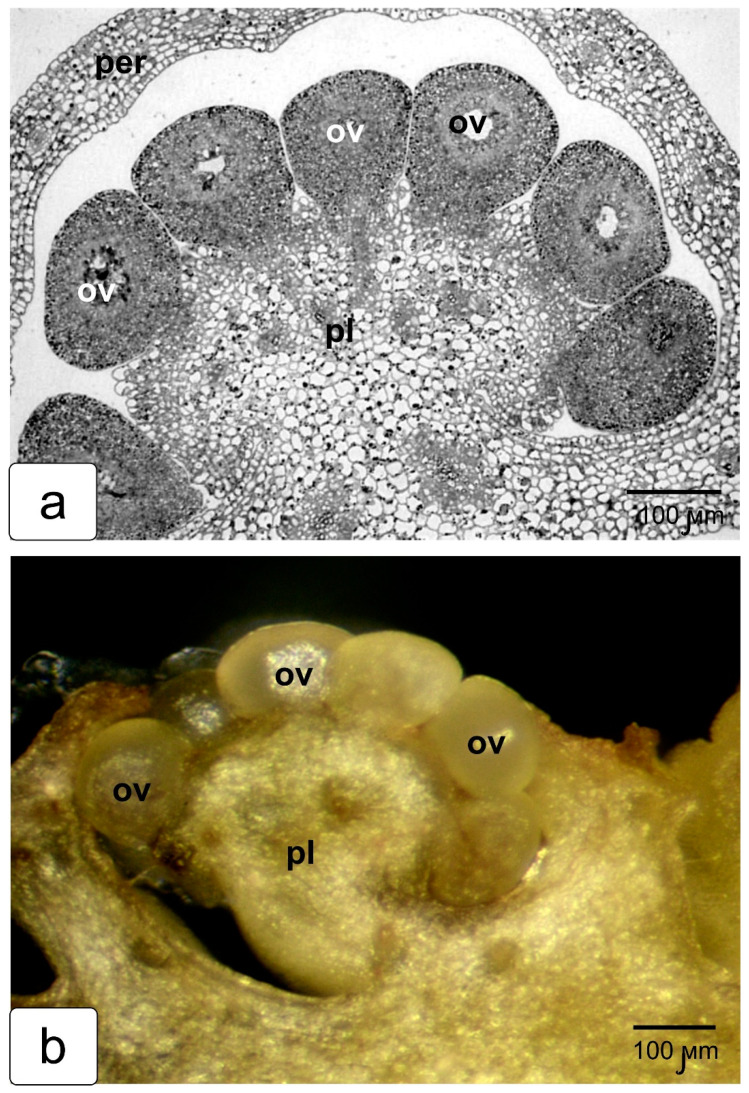
Locular cavity (seed chamber) of a tomato fruit at an early (initial) stage of development: transverse anatomical section (**a**); locular cavity of a fruit of about the same age after manual dissection (**b**). Designations: ov—ovule; per—pericarp; pl—placenta.

Figure 3 shows a transverse anatomical section of the seminal chamber of a tomato fruit at an early stage of development (a) and a photograph of the placenta with ovules from a freshly opened fruit of the same stage of development (b) in order to have a three-dimensional idea of this stage of fruit development.

**Figure 4:**
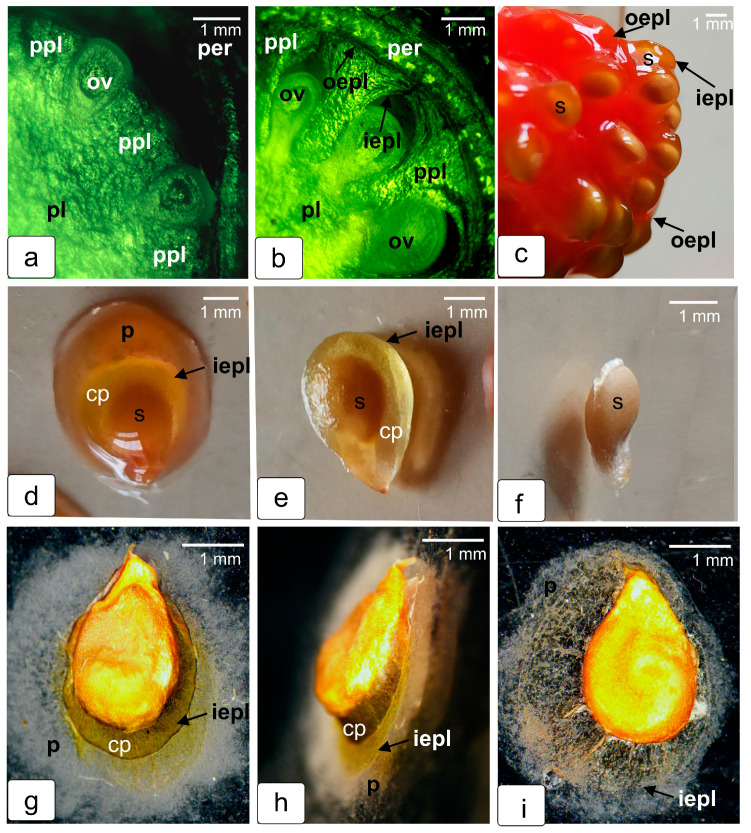
Photographs of freshly extracted internal tissues from tomato fruits. Cuts in the early stages of development: the beginning of parallel growth of ovules and protrusions of placenta tissue (**a**); capsules formed around the ovules (**b**); seeds taken out with gel from a mature tomato fruit (**c**); capsule with a seed and a layer of gel on the surface, extracted from a red fruit (**d**); seed capsule with gel removed (**e**); seed, pulled out of seed capsule (**f**). Seed capsules taken from a ripe fruit and dried on a glass: an intact capsule is stretched over the seed due to the air in it (**g**,**h**); the previously pierced seed capsule was completely spread on the seed and on the glass (**i**). Designations: cp—seed capsule; oepl—outer epidermis of protrusions of placenta tissue; iepl—inner epidermis of protrusions of placenta tissue; ov—ovule; per—pericarp; pl—placenta; ppl—protrusion of placenta.

The following stages are shown on fragments of transverse sections of the next stages of fruit development. Figure 4a shows how the ovules sink into the tissue of the growing placenta, which then completely surrounds them. As a result, the ovules are inside a completely closed capsule (Figure 4b). In a ripe red tomato fruit, the capsules with seeds are immersed in a jelly-like mass of released parenchyma cells that have lost contact with neighboring cells of the protruding placenta tissue (Figure 4c). They are held together only by a thin shell—outer epidermal tissue of placental protrusions, carefully pushed away from the pericarp when extracted from the fruit. In order to demonstrate the tightness of the seminal capsule, the capsule was carefully removed from this jelly-like mass, along with a portion of the gel on the surface (Figure 4d). The capsule was then carefully released from the adhering mass (Figure 4e), and after cutting, the seed was carefully removed (Figure 4f). In addition, one of the capsules just removed from the fruit was placed on a glass slide and left to dry completely (Figure 4g,h). It is clearly seen that the capsule shell stretched after drying, retaining the internal air space, which can serve as proof of its complete tightness. In another capsule, the impermeability was broken by piercing it, and it was also placed on a glass slide until dry. This capsule, when dried, completely spread out on the glass (Figure 4i). The obtained data clearly demonstrates the fact that the development and maturation of the seed in the tomato fruit occurs inside the capsule in complete isolation from the surrounding tissues.

**Figure 5:**
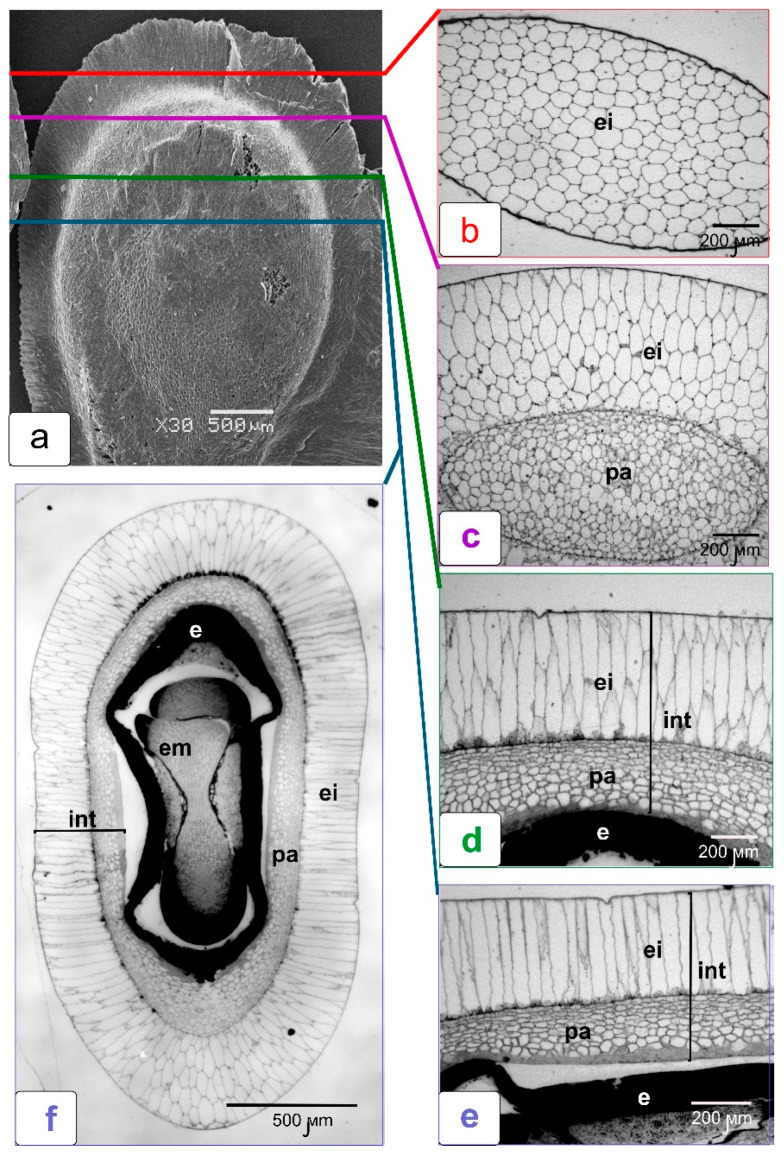
The light microscopy of the seed structure from a close-to-ripening green tomato fruit on cross sections. Sectioning locations (instead of a diagram) are shown in the seed image taken on scan (**a**). Transverse semi-thin sections of seeds (light microscopy) extracted from a ripening tomato fruit in different zones: a section passing through the epidermis of the integument in the upper part of the seed (**b**); a section passing through the parenchyma cells and epidermis of the integument (**c**); fragment of a transverse section, including the integument and tightly adjoining endosperm tissue (**d)**; a fragment of a transverse section, including the integument and loosely adjoining endosperm tissue (**e**); a cross section of tomato seed in the embryo zone (**f**). Designations: e—endosperm; em—embryo; ei—outer epidermis of integument; int—integument; pa—parenchyma of integument.

Tomato seeds in their structure have features in which they differ markedly from the seeds of most other *Solanaceae* and, first of all, in the structure of the seed coat (skin). The process of formation of the tomato seed coat at the ultrastructure level was studied in detail in our previous article [10]. In this study, we examined in more detail the outer layer of the seed coat and the features of its interaction with the shell of the seed capsule (Figure 5). The outer epidermis of the seed coat consists of long, elongated (giant) cells that form a kind of crown around the seed. At first glance, this crown borders the seed only along the edge (Figure 5a). However, anatomical sections of the tomato seed show that the epidermal cells evenly cover the entire seed (Figure 5f). The transverse section of these cells (upper end of the seed) shows that they have a multifaceted shape and are heterogeneous in the size of their transverse profiles (Figure 5b). On longitudinal sections (in the lateral parts of the seed), these cells have the same length but differ by cell area (Figure 5b–e).

**Figure 6:**
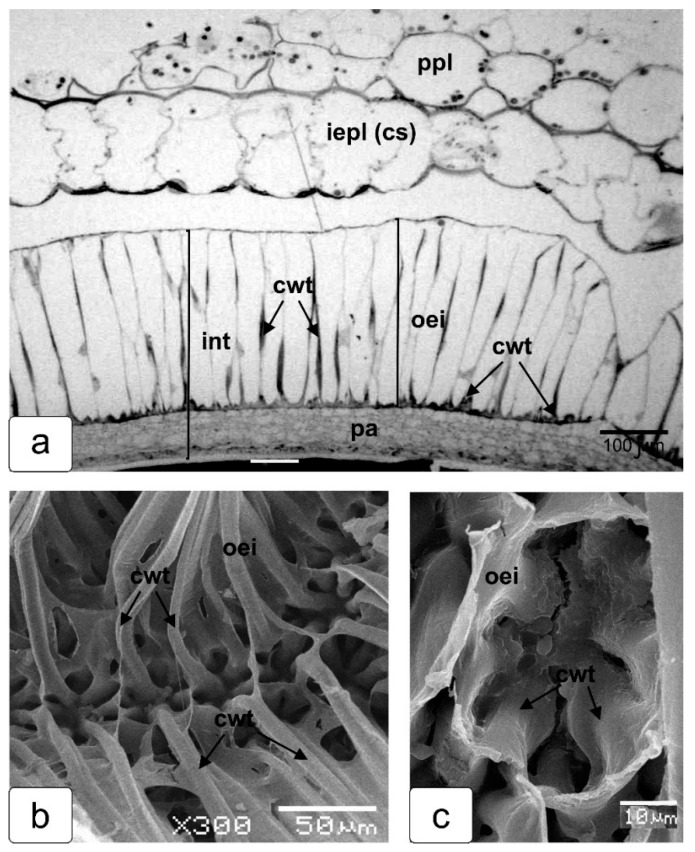
Fragment of an anatomical cross section of the tomato seed coat, together with part of the shell of the emerging seed capsule. The walls of elongated cells, which forming the coat outer layer of a mature seed have thickenings at the base, turning into local thickenings, a kind of cell wall, which can be traced throughout the cell stretch (**a**). After the seed dries up and is released from the capsule, these elongated cells crack in extended sections of cell walls (having no secondary cell walls) between these "ribs" into separate ribbons (**b**). Thickened fragments formed by deposits of the secondary cell wall are clearly visible at the base of each cell and other surfaces (**c**). Designations: cs—capsule shell (iepl—inner epidermis of the protrusion of placental tissue); cwt—cell wall thickening; oei—outer epidermis of integument; ppl—protrusion placenta tissue.

Figure 6 shows a fragment of a cross section of the seed coat from a green (before reddening) tomato fruit together with a fragment of the protrusion of placental tissue which, as the tomato fruit ripens, acquires the structure of the pulp. The beginning of separation from the epidermis and the placental parenchyma breaks up into separate cells and forms a pulp in a mature fruits, maintaining the integrity provided by the outwardly emerging inner epidermis of the placental protrusion (iepl), which is visible (Figure 4c). The epidermis of the protruding placenta tissue develops into the shell of the seed capsule (cs). The outer and inner walls of a thin layer of the epidermal cells of the placental protrusion (iepl) have significant thickenings, which probably provide strength and a kind of independent development of this tissue and the ability to form a capsule (cs). Significant thickening of the cell walls of the outer epidermis of the seed integument (cwt) is also noticeable, and is especially pronounced in the lower part and along the entire length of the cells. These parts of the cell walls of the seed coat are markedly fragmentary thickened (cwt) compared to the outer cell wall (Figure 6a). At this stage of the tomato fruit maturation, the cell walls of the outer layer of the seed coat at the base and in other parts of the walls thicken by lignification [10]. After drying and release from the seed capsule, the elongated cell faces towards the outer layer of the coat crack along the ribs into separate ribbons. This is clearly seen in the image obtained from scanning electron microscopy (Figure 6b). Thickened cell walls are clearly visible at the base of each cell (Figure 6c).

**Figure 7:**
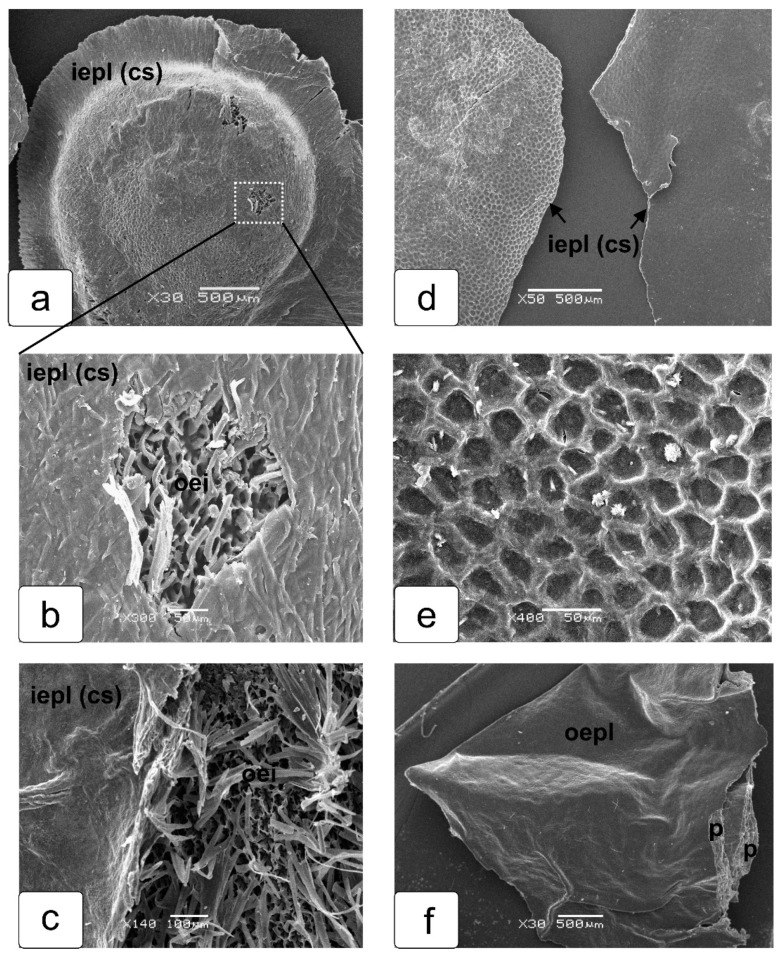
Details of the structure of the shell of the placenta seed capsule (formed by epidermal cells of the protruding placental tissue) of the tomato fruit, revealed from scanning electron microscopy. The capsule shell was removed from the dried seed together with the cell fragments of seed epidermal cells (**a**–**c**). The inner side (adjacent to the seed) of this shell (**d**,**e**) and its outer side (**e**,**f**) are shown. On the inner side, the end walls of the outer layer cells of the seed coat were imprinted (remained) (**e**). The outer side of the outer epidermis of the placental protrusion looks elastic and quite strong and retains desiccated fragments of the placental protrusion (**f**).

Features of the structure and the structure of the single-layer shell of the seed capsule are shown in Figure 7. The fragment seed capsule shell (representing the epidermis of the placental protrusion) was removed from the dried seed together with the some parts of the outer epidermis of the seed, since separation of these tissues was difficult during drying (Figure 7a–c). Its inner (adjacent to the seed) and outer sides (Figure 7d,e) are shown. On the inner side, the end walls of the cells of the outer layer of the seed coat were imprinted (remained) (Figure 7e). This indicates that these cells of the epidermis of protrusion of the placental tissue retain their structure and shape, which ensures the integrity of the seed capsule. The outer side of the seed capsule shell is rather smooth (Figure 7d,f), and it also looks elastic and quite strong (Figure 7f).

**Figure 8:**
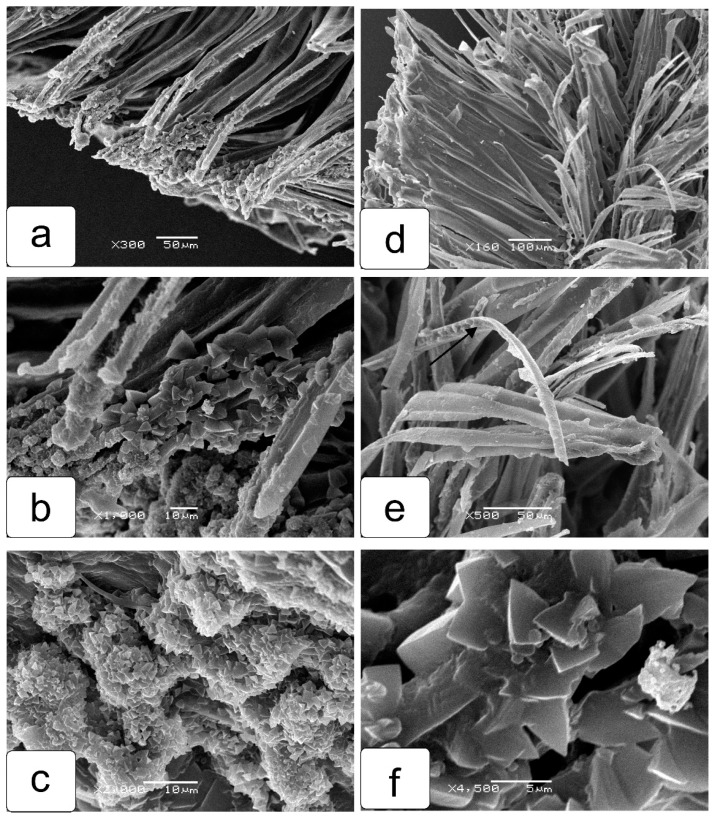
Calcium oxalate crystals in the cells of the outer layer of the seed coat. After opening the seed capsule, the cells begin to break into ribbons from the cell walls (**a**,**b**,**d**,**e**). A large number of calcium oxalate crystals (**a**–**c**,**f**) accumulate on the walls themselves and at their ends.

Further study of the tomato seed, dried together with the seed capsule, revealed a very important detail which made it possible to understand how the cells break into separate ribbons. When the seed capsule shell was removed, only bursting cells were found, and accumulations of calcium oxalate crystals were observed inside and especially at the ends of the formed ribbons (Figure 8a–c,f). Apparently, the crystals accumulate in these cells gradually during the development of the seed. It is quite obvious that the crystals play a major role in the tearing mechanism of the cells of the outer layer of the seed coat into ribbons.

**Figure 9:**
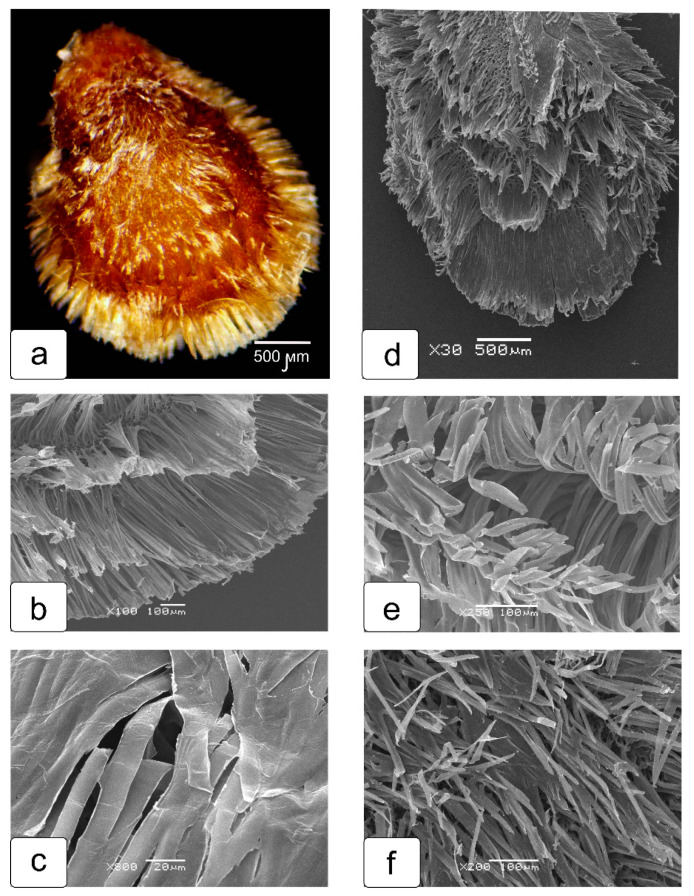
Stages of seed coat formation with ribbon-like hairs. Seed removed from the seed capsule and washed (**a**). The same seed on a scanning electron microscope (**d**). Gradual divergence (fluffing) of the cell walls into separate ribbons (**b**,**c**,**e**), which then take the form of hairs (**f**).

However, when examining the surface of the seed removed from the seed capsule and washed (Figure 9a), no crystals are observed. The bursting cells of the seed coat first form rows of different configurations and density from the walls (Figure 9b,c), and then gradually separate from each other. Their ends are slightly twisted upon further drying (Figure 9a,f). On the surface of a dry seed, they look like hairs (Figure 9a).

## 3. Discussion

The structure of the seed coat of any plant is related to its protective function [11]. The thickness and number of layers in the seed coat depend on the type of ovule [2]. However, the features and shape of the outer layer of the seed coat largely depend on the physiological conditions inside the fruit in which this seed develops. It is known that the outer epidermis of any plant organ serves to protect against an unfavorable external environment. Therefore, the structure of the epidermis must be adapted to the environment in which development takes place [12,13].

In our previous work, the structural features of the formation of the tomato seed coat at different stages of development were studied and analyzed in detail, and the degree of participation of each tissue in this process was shown [the number is changed]. The outer and inner epidermis of the only integument of the tomato unitegmic ovule was found to take the main part in the formation of the tomato seed coat. In the process of differentiation, the cells of the outer epidermis are strongly elongated perpendicular to the surface of the seed, and their walls at the base thicken due to lignification. At the same time, they maintain the internal structure of living, functioning cells. As a result, a strong outer layer of the seed coat is created. And the cells of this layer in mature tomato seeds split into separate “hairs”. There is a wide variety of tomato seeds, the coat structure of which may have some changes due to mutations and genetic deviations. However, the manner of hair formation (pubescence) on the surface of the tomato seed remains unchanged, except for tomato fruits with hairless (smooth) seeds [14]. Previously, the formation of hair-like structures from the cells of the outer layer of the seed coat was studied in a number of representatives of the *Solanaceae* family [15]. It has been suggested that these structures in *Solanum nigrum* are associated with cracking of the thin cell walls “pores”. The authors considered that the putative epidermal cell wall structure with lateral strands of thickening arising from pyramid-shaped bases, and connected by unthickened membranes is the “pore”. However, in our opinion, the authors made an erroneous assertion from the obtained results of the scanning electron microscopy.

We are interested in the reason for such an unusual structure of the outer layer of the tomato seed. Considering that tomato seeds are formed in a humid environment inside a juicy fruit, one could assume that this circumstance was the reason for such an unusual differentiation. However, it was not clear how such membranes could protect against the surrounding moisture, especially since these cells are later torn into hairs. It remained to find out what the mechanism was that underlies this process.

The tomato fruit is a convenient object for various biochemical or molecular studies of the development of fleshy fruits. In such works, some anatomical data are given on the various tissues that form the tomato fruit. But the issue of the formation and structural organization of the tissue in which the ovules are immersed has not been sufficiently studied [7]. Most of the studies have discussed its supposed role in the non-germination of seeds in the moist environment of a juicy fruit. Only occasionally was the so-called “mucous shell” around the growing seed mentioned, but without anatomical data that would demonstrate this shell [9]. It is generally accepted that the seeds inside the juicy fruit of a tomato in an overripe form are in a locular cavity filled with cell sap from the maceration of cells and the converting of walls of the placenta cells into mucilage. This gel-like liquid is believed to contain substances that inhibit seed germination [7,16].

Our detailed anatomical study of different development stages of the tomato fruit made it possible to understand how the protrusions of placental tissue are formed inside each seed chamber (locular cavity) of the tomato fruit and how, as a result of this process, individual capsules for each seed are formed. The process of formation of protrusions of placental tissue occurs synchronously with a development of ovules in the seed chambers of the juicy fruit. On a transverse anatomical section of an early fruit, placental outgrowths look like tubercles of small cells covered with epidermis (Figure 2a–d). But if we imagine this in the volume of the whole fruit, then these outgrowths will turn out to be bolsters surrounding the ovule from all sides. As the placenta grows further, it completely encloses and immerses each developing seed in its own isolated seed capsule (Figure 2d–f). The upper epidermises merge into the common epidermis of the multicapsular placenta at the border with the inner epidermis of the pericarp and come into contact with it. As the tomato fruit ripens, the parenchyma cells of the placenta are detached from each other, they lose contact with neighboring cells, become mucilaginous and convert into a so-called gel. But the single-layer epidermis of the placenta that surrounds each seed is preserved and becomes the shell of the seed capsule. Despite the rather thin cell walls, this tissue turned out to be quite strong and elastic, capable of ensuring the impermeability of the seed capsule, which was tested in various ways (Figure 4). This is confirmed by the study of the capsule shell due to scanning electron microscopy (Figure 7). The data obtained in the work allow us to conclude that the tomato seed does not come into contact with the destroyed cells of the placenta, but rather is formed in a dry air environment of a hermetically sealed seed capsule. The details of the formation of seed capsules in the juicy fruit of tomato are described in our study for the first time. There are no analogues of the description of the formation of such a structure in the references known to us.

The accretion of the growing ends of any tissue is a common phenomenon during the formation of embryonic structures [1,17]. For example, the histogenesis of the ovule of almost any plant is associated with the synchronous (correlated) growth of the nucellus and its surrounding integuments. Formed integuments at the junction first form a pollen micropyle, and after fertilization of the embryo sac in most cases, they merge. In addition, when studying the phylogenetic issue of the origin of double-integumentary and single-integumentary (bitegmic and unitegmic) ovules in flowering plants, the prevailing opinion is that the only integument in unitegmic ovules was formed by the merger of two integuments [18].

The data obtained also led to the conclusion that the peculiar structure of the outer layer of the tomato seed coat is, first of all, necessary to support the seed capsule shell and maintain optimal air volume, since seed respiration is extremely important for viability.

In the present work, using scanning electron microscopy, another important issue was examined: how the splitting of elongated cells of the outer layer of the seed coat occurs. It was revealed that a large number of calcium oxalate crystals are synthesized and accumulated in these cells (Figure 8). Opening, most likely, occurs according to the same type as in tomato anthers [19,20,21]. For tomatoes in particular, as well as for the entire *Solanaceae* family and many other plants, the formation of calcium oxalate crystals is well studied [22,23,24,25]. Therefore, oxalates are found in specialized leaf cells, ideoblasts, in the cells of the crystalline anther parenchyma [20,21]. However, the presence of crystals in the epidermal cells of the coat of the tomato seed has not been shown previously. This omission may be due to the fact that when seeds are isolated, it is rarely possible to preserve the seed capsule and avoid moisture on the surface. Thus, the detection of crystals can be carried out only by drying the fragments of the fruit without damaging the epidermal tissues of the placenta, which form the protective individual capsules of the seeds. We believe that the participation of calcium oxalate promotes a specific rupture of cell walls into identical strips, which then twist at the ends, acquiring the appearance of hairs.

The cumulative development of plant tissues is accompanied by the coincidence of many developmental patterns in both seed tissue and fruit tissue [26]. A particular case of co-regulation of such developmental processes can be the formation of the seed coat, accompanied by the specific death of part of the epidermal cells [9]. The evolutionary process may have ensured the selection of forms with expanding tomato placenta, providing greater attraction for fruit-eating animals, which increased the plant’s distribution areal. Thus, for plants that form succulent fruits, it is known that birds and small mammals are their consumers, which required, in the course of evolutionary development, the prevention of immediate seed germination for better distribution of seed propagules [27]. 

## 4. Materials and Methods

### 4.1. Plant Material

In this study, a tomato (*Solanum lycopersicum* L.) cv. Beliy Naliv, a well-studied model variety with non-cracking, juicy fruits, was used. Tomato plants were grown from seeds using seedlings until fruiting within 130 days. Each plant was cultivated in a 5 L pot filled with a special substrate for growing tomatoes, a Biolan Growing Bag (Eura, Finland). The cultivation was carried out under the following environmental conditions: a light/dark cycle 16/8 h, a temperature of 24/20 °C, a light intensity 200 μmol m^−2^ s^−1^, and relative humidity 60%. To ensure fertilization, the flowers were shaken after full opening. The ovaries and developing succulent fruits were taken at different periods of ovule development from the first day after fertilization to full fruit ripening (50 days). Special attention was paid to the beginning of the expansion of placenta tissue from the appearance of protrusions to complete closure of the placenta epidermis and the formation of capsules around the seeds. 

### 4.2. Fixation and Sample Preparation

Isolated ovules at different stages of development were fixed with 2.5% glutaraldehyde (Merck, Darmstadt, Germany) in 0.1 M Sorensen’s phosphate buffer (pH 7.2) containing 1.5% sucrose, cooled to +4 °C for 24 h., washed twice with a chilled buffer, and then fixed in 1% OsO_4_ (Sigma-Aldrich, St. Louis, MO, USA). Dehydration was carried out in increasing concentrations of ethanol (30, 50, 70, 96 and 100% for 30 min each). Further, propylene oxide was used for pouring into the resin and poured into a mixture of Epon-812 and Araldite (Merck, Darmstadt, Germany) according to a standard protocol. The ovary fragments were then moved into containers with the addition of a catalyst, oriented for the convenience of subsequent cutting, and subjected to a two-stage polymerization. Light-optical analysis was performed using semi-thin sections obtained using an LKB-V ultramicrotome (LKB, Bromma, Sweden). Sections (1–2 μm) were placed in a drop on glass slides, dried, stained with 0.1% methylene blue solution (Merck, Darmstadt, Germany), and washed with distilled water. Prepared permanent preparations were placed in epoxy resin under a glass lid. Samples were analyzed using an Olympus BX51 microscope (Olympus, Shinjuku, Tokyo, Japan). To obtain digital images, a Color View II camera (Soft Imaging System, Muenster, Germany) with the Cell program was used. Over 300 samples of the above ovule tissues were analyzed from three independent tomato plants.

### 4.3. Sample Preparation for TEM Analysis

For electron microscopy, the same preparations of ovules embedded in an epoxy-araldite composition were used. Ultrathin sections were made using a 1.5 mm wide diamond knife on an LKB-V ultramicrotome (LKB, Bromma, Sweden). Sections were placed on mixtures or grids coated with formvar and contrasted with solutions of uranyl acetate and lead citrate according to the standard method. Thin sections were then analyzed and photographed using an H-500 (Hitachi, Ibaraki, Japan) and JEM-1400 (Jeol, Akishima, Tokyo, Japan) electron microscope.

### 4.4. Seed Analysis by Scanning Microscopy

Mature tomato seeds were dried and placed on an SEM plug covered with adhesive tape (double sided carbon tape, 8 × 20 mm, Shanghai, China). The samples were then coated with gold and palladium using an Eiko IB-3 ion plating machine (Eiko, Tokyo, Japan). The thickness of the coating layer was 20 nm. The preparations were then analyzed at a working magnification of ×30 to ×5000 under Camscan-S2 SEM (Cambridge Instruments, Cambridge, UK) in the Laboratory of Electron Microscopy (Faculty of Biology, Lomonosov Moscow State University). 

## 5. Conclusions

For the first time, the presence of a specific structure formed by the epidermis of placental protrusions that provides a unique system of seed encapsulation and creates a protective film around the placenta that transforms into pulp in the later stages of ripening was described in detail. Due to the encapsulation, the seed develops in the air environment and does not come into contact with the surrounding cells of the collapsing placenta. It is concluded that this is why there is no premature germination of seeds in overripe tomato fruits. The unique outer layer of tomato seeds, consisting of elongated and lignin-reinforced cells, creates a kind of framework that apparently provides the necessary air volume inside the seed capsule. In addition, the unusual structure of the cell walls of this tissue leads to the formation of a hairy coating of the seed as a result of cracking of the cell walls along the entire length of these cells with the formation of ribbons. The mechanism of this cracking, which is similar to a process in tomato anthers, was identified and discussed in the study. This became possible due to the fact that accumulations of calcium oxalate crystals were found in the inner cavity of the cells of the outer layer of the seed coat.

## Data Availability

Not applicable.
